# What Is the Relation between Circular Economy and Sustainability? Answers from Frontrunner Companies Engaged with Circular Economy Practices

**DOI:** 10.1007/s43615-021-00064-7

**Published:** 2021-06-02

**Authors:** Anna M. Walker, Katelin Opferkuch, Erik Roos Lindgreen, Andrea Raggi, Alberto Simboli, Walter J.V. Vermeulen, Sandra Caeiro, Roberta Salomone

**Affiliations:** 1 Deptartment of Economic Studies, University “G. d’Annunzio”, Pescara, Italy; 2 Department of Science and Technology, Universidade Aberta, Lisbon, Portugal; 3 Center for Environmental and Sustainability Research (CENSE), NOVA University, Caparica, Portugal; 4 Department of Economics, University of Messina, Messina, Italy; 5 Copernicus Institute of Sustainable Development, Utrecht University, Utrecht, the Netherlands

**Keywords:** Circular economy, Sustainability, Semi-quantitative survey, Semi-structured interviews, Mixed methods, Private sector

## Abstract

The circular economy (CE) concept has become a major interest for companies, promising new business opportunities and a decrease in environmental impacts. Though research on circular business models has recently increased, few scholars have investigated how companies engaged with CE view the connection between CE and sustainability. To address this gap, this paper uses a semi-quantitative survey and semi-structured interviews conducted with companies based in Italy and the Netherlands. Purposive sampling was employed to target firms associated with national and international CE networks, as these companies already engage with CE practices. The survey was distributed online to over 800 firms, of which 155 provided information on their understanding of the CE concept and its relationship with sustainability. The survey results are complemented through findings from 43 interviews with a subset of the survey respondents. The survey answers show that companies view CE as one of the tools to achieve sustainable development, particularly in the environmental domain, where the focus lies on environmentally friendly resource use. Yet, the respondents are less confident whether CE increases economic and social benefits of firms. Interviews show that a majority of respondents position sustainability as the overarching concept. However, most companies advocate that the private sector should strive for both sustainability and circularity, though the distinction between the two concepts in daily business operations seems synthetic and futile to some. These findings provide an important stepping stone for better understanding how firms could apply CE practices to move towards a more sustainable society.

## Introduction

Companies are increasingly engaging with the concept of circular economy (CE) [1], further integrating CE practices within organisational sustainability strategies. As the definition and possibilities of CE evolve, so does its relation with sustainability, requiring a re-clarification of the two concepts. Sauvé et al. [2] pointed out that the transdisciplinary nature inherent to sustainable development (SD) results in difficulties formulating one single conceptualisation. This is because of the intermeshing of diverse disciplines, vocabularies and stakeholders. Some authors suggest the interpretive flexibility of sustainability is a strength, as it enables the concept to be adapted to a variety of contexts and institutions [3]. Others argue sustainability’s vagueness hinders operationalisation [4]. Either way, efforts have been made to find a consensus on SD’s conceptualisation [5] based on the globally accepted Sustainable Development Goals (SDG) framework [6]. In many ways, the ambiguity of SD can be extended to the concept of CE [7, 8]. This reality has encouraged numerous scholars to perform literature reviews in an attempt to improve the clarity of CE as a concept [9–12].

Similarly to SD, CE can also be considered an umbrella concept, drawing inspiration from a diverse set of resource management concepts and ideas from environmental sciences that were popularised in the 1960s [13]. While numerous CE strategies exist and discussions on the impact of such strategies are increasing [14], the core of CE can be described by its aim to retain value through the process of closing resource loops [15]. In some academic literature, this description has evolved, with numerous discourses suggesting CE can reduce harmful environmental impacts, stimulate economic growth and more recently, generate positive social impacts [16, 17]. Nonetheless, several authors have also shown that “circular” practices do not always result in sustainable impacts, potentially leading to sustainability trade-offs [11, 18, 19].

Given companies play a key role in the transition to a CE [20], their interpretation of the relation between CE and sustainability will provide insights into decision-making processes concerning the previously mentioned trade-offs and might reveal to what extent they consider their CE practices help to solve current sustainability issues. However, company perceptions of this relation have previously been overlooked in CE literature. Various articles study drivers for implementing CE solutions at the company level (e.g. [21]), but do not investigate the explicit question of how the concepts are connected. In addition, it has been noted that empirical research on larger numbers of cases is still uncommon in CE literature [22]. Furthermore, other studies have indiscriminately surveyed companies regardless of their engagement with CE [1, 21, 23], making it difficult to distinguish between the perspectives of companies that are engaged with CE practices and those which are not.

Therefore, this article aims to empirically explore how frontrunner companies engaged with CE practices understand the connection between CE and sustainability*.* In this research, frontrunner companies are considered as private sector organisations which are early adopters of CE practices and are involved in cross-sectoral networks to exchange experiences and further their knowledge on CE. For both reasons, it is assumed that they possess considerable insights on the topic [24]. To attain the research aim, four objectives were formulated to identify how companies engaged with CE: (a) describe CE, (b) describe sustainability, (c) describe the link between CE and sustainability, and (d) prioritise these two concepts.

An explorative mixed-methods approach consisting of a semi-quantitative survey and semi-structured interviews was implemented to enquire companies’ perspectives on the connection of CE and sustainability. The companies selected within this study operate in either Italy or the Netherlands. Both are leading European countries in publishing CE research and implementing CE practices [23, 25]. Moreover, both countries have established national and regional level CE networks, resulting in an ecosystem of companies involved with the development of CE [26]. With no restrictions on company size or industry sector, a diverse range of perspectives were uncovered to reflect the experiences of companies integrating CE practices, while progressing towards sustainability goals. The remainder of this article presents a literature review (“Academic Perspectives on the Relation between CE and Sustainability” section), the mixed methods approach employed (“Methods” section), results obtained for each research objective (“Results” section), their discussion and synthesis of the overall research aim (“Discussion” section) and concluding remarks (“Concluding Remarks” section).

## Academic Perspectives on the Relation between CE and Sustainability

A vast array of conceptual interpretations of both CE and sustainability exists within CE-related literature [7, 8]. In fact, positioning CE in relation to the more established concept of SD has become a dominant topic of discussion [2, 10, 17, 18, 27, 28]. Sauvé et al. [2] contrast CE with environmental sciences and SD, noting that CE provides a relatively clear “angle of attack” to solve environmental problems. The multitude of relations is also addressed by Geissdoerfer et al. [10], uncovering three general groups of relationships: a conditional- (CE as a condition for SD), beneficial- (CE benefits SD) or a trade-off- (CE having both positive as well as negative sustainability impacts) relationship. In their literature analysis, Schöggl et al. [28] highlight that CE solutions can also carry negative sustainability outcomes, due to e.g. rebound effects (see also: [19]). They state that social topics remain underrepresented in CE and that higher-ranking value retention options, with potentially higher sustainability impacts, are less clearly addressed in CE literature. Next, Schroeder et al. [17] identify the extent to which CE practices are relevant for the implementation of the SDGs. The links between CE and the different SDGs range from weak/non-existent (e.g. SDG 3 on Good Health and Wellbeing and SDG 5 on Gender Equality) to strong/direct (e.g. SDG 8 on Decent Work and Economic Growth and SDG 12 on Responsible Consumption and Production). Lastly, using a critical literature review and timeline of CE conceptualisations, Calisto Friant et al. [13] develop a typology of circularity discourses. They present a conceptual differentiation between the *Circular Economy discourse*, which primarily offers a technical perspective of material efficiency, and the more SD-related *Circular Society discourse*, which also includes the redistribution of wealth, knowledge, technology and power throughout society.

While the academic debate on the relation between CE and sustainability is lively, the perspective of companies active in CE seems overlooked in CE literature. Yet, this perspective potentially carries insights about their envisioned contribution to solving current sustainability issues through the real-world impacts of firms’ CE solutions. Several studies analyse drivers and barriers for implementing CE solutions at the company level (e.g. [21, 29]), but do not explicitly address how the concepts CE and sustainablity are related. Brown et al. [24] investigate why companies engage in CE collaboration and interestingly find that the actors’ motivations are rooted in normative values for sustainability. However, the participants’ interpretation of the connection between CE and sustainability is not assessed. Related thereto, Ritzén and Sandström [30] ask manufacturing companies about barriers to CE and find that the lack of integration of different domains, such as sustainability and CE, forms a barrier to the CE transition. In addition, the definition of the concepts might potentially be context-dependent, as is the case for one of the underlying fields of study of CE, namely industrial ecology. Deutz et al. [31] have shown that the understanding and manifestation of industrial ecology approaches such as industrial symbiosis can vary considerably, both within and amongst countries. This context-dependency of the definition has not yet been analysed for companies with CE practices.

Furthermore, no consensus exists with respect to how two emerging aspects, bioeconomy and sufficiency, are relevant within these discussions about the relation between CE and sustainability. Bioeconomy has been considered a possible “sustainability avenue” [32], and its contribution to CE has been investigated [33]. The regenerative nature of biological materials, in which output materials can be returned to the cycle through processes such as composting, is in line with the circulation of resources in a CE [33]. Still, whether this concept is integral, complementary, or an alternative to implementing CE remains unclear [34]. For sufficiency, the possibility of realizing a CE in a world with growing consumption rates has been scrutinised by some [18, 35]. To address this, recent studies have proposed a further paradigm shift towards a sufficiency-based CE [36]. Introduced as a characterising feature of CE by Stahel [37], sufficiency is also described as a new paradigm within industrial sustainability. It takes a societal-wide focus on reducing consumption, evolving from lean manufacturing, cleaner production and CE [36]. Compared to the earlier understanding by Stahel [37] which was mainly based on the reduction of waste through value retention, Bocken and Short [36] also underline that sufficiency prevents rebound effects and thus entails an absolute decrease in consumption. Furthermore, consumers and policy makers have a larger role to play in sufficiency than within the previous paradigms, in which the market and technology were seen as the main levers. However, whether frontrunner companies engaged with CE practices are aware of both this paradigm shift and the role of bioeconomy in this transition remains unexplored.

Lastly, the promotion of CE as a tool to positively influence all three dimensions of sustainability of an organisation, as popularised by the Triple Bottom Line (TBL) concept [38], often ignores the dilemma of sustainability trade-offs [11]. It is important to note that such “CE trade-offs” would alter the interpretation of the relation between CE and SD and substantiate the necessity of accurately assessing the effects of CE solutions before their implementation. The blurry boundary between CE and sustainability and lack of insight into company-level interpretations of the two concepts, ultimately, constrains the efficacy of organisations implementing CE to contribute towards reaching the SDGs.

## Methods

This section describes the mixed-methods approach [39] employed within this study. The authors opted for semi-quantitative and a qualitative research method, which are mainly applied to research of an explorative nature [40]. The first method was a semi-quantitative survey [39], which does not only focus on the frequency of respondents’ characteristics within the sample but also analyses the variety of these characteristics. The second method employed were semi-structured interviews [41] to better understand why and how companies connect the concepts of CE and sustainability. The following paragraphs describe the individual steps of the method, starting with the sampling procedure (“Sampling Procedure “ section). The obtained sample is then presented in the sample description (“Survey Development” section) and the types of questions asked are documented in the survey development (“Survey Development” section). After collecting the survey answers, the interview process (“Interview Process” section) was developed and both data sources integrated into the iterative data analysis (“Data Analysis and Integration” section).

### Sampling Procedure

A purposive sampling method [42] was applied to identify companies actively engaged with CE practices in Italy and the Netherlands. Though this sampling method reduces the potential target population for sampling, it increases the possibility that the whole sample has specific characteristics (i.e. possessing insights on CE) that are desirable to address the research question. Despite CE continuing to grow in popularity, the number of self-identified “circular firms” is limited [43]. Hence, the authors focused the sample on companies within existing national and international CE networks depicted in Table 1, as these firms were assumed to be frontrunners in conceptualising and engaging with CE practices [24]. CE experts, involving policy makers, university professors and CE network coordinators in the Netherlands and Italy, were consulted to ensure representative coverage of CE networks, thus avoiding a sampling bias, which could result in the exclusion of relevant CE networks [44]. Furthermore, to minimise the coverage error occurring if companies are missing within the sampling frame, the researchers consulted updated network member lists online or directly contacted the CE network coordinators.

**Table 1:** Sampling protocol and data collection overview

CE networks considered	● Atlante Italiano dell’Economia Circolare (IT) ● Italian Circular Economy Stakeholder Platform (ICESP) (IT) ● Circular Economy Network (IT) ● Mercato Circolare (IT) ● Circulair ondernemen (NL) ● Ontertekenaars van Grondstoffakkoord (NL) ● Circle Economy (NL) ● Holland Circulair Hotspot (NL) ● Circulaire Coalitie (NL) ● Ellen MacArthur Foundation CE 100 (international) ● Circular Economy Club (international)
Inclusion criteria for companies	● Part of a local or international CE network listed above ● Primary business operations in either NL or IT, if member of international networks ● Legal form is a private sector organisation according to national law ● Online presence through an official website
Survey delivery and responding period	● Delivered online via Survey Monkey, with personalised email invitation and customised links ● Three reminder emails sent out within intervals of 3 weeks ● Three months total responding period: July–September 2019
Interviewing process	● Invited companies that indicated their availability for interview within the survey ● Sent out interview guidelines at least 1 week prior to interview ● Conducted semi-structured interviews through video calls ● Interview period: May–June 2020

At the end of the survey, respondents were asked to voluntarily opt-in for the subsequent interviews with the researchers; thus, the interview sample constitutes a subset of the survey respondents. The answers to both the survey and the interviews were anonymised to ensure the establishment of participants’ trust and additional insights on participants’ experiences.

### Sample Description

The survey was sent out online to a total of 809 companies and was completed by 171, of which 155 responses were valid. This represents a survey response rate of 19%, which is considerable for business surveys [45]. From these 155 respondents, 46% were based in Italy and 52% in the Netherlands. Two respondents were part of Italian or Dutch CE networks while being based outside of these countries: one from Luxemburg and one from Austria. Similarly, in the interviews, the distribution of firms (*n* = 43) was nearly the same, with 20 companies based in Italy and 23 in the Netherlands. This almost equal distribution in both the survey and interview sample reduces the risk of country bias in the results.

According to the Eurostat classification scheme for Small and Medium Enterprises (SMEs) [46], around 45% of the survey respondents were micro-companies, as depicted in Table 2. Concerning the interviewees, almost half of them were also micro-companies, while the rest was equally divided into SMEs and large companies. From Table 3, it becomes evident that the survey has reached both decision-makers who have management-level responsibilities, as well as employees that are closely involved with sustainability and Corporate Social Responsibility (CSR) activities. In a similar vein, most interviewees were from General Management, followed by the Sustainability and CSR department. However, the overall sample share of respondents from these two departments was larger, indicating a higher propensity of these professionals to be interviewed. The inclusion of respondents influencing companies on a strategic level supports the credibility of the provided information in both the survey and the interviews.

**Table 2:** Company size of survey and interview respondents

Company size	Number of employees	Survey		Interviews	
		Respondents *(n = 155)*	Company size (*subtotal)*	Respondents *(n = 43)*	Company size *(subtotal)*
Micro companies	1 to 9	45%	45%	49%	49%
SMEs	10 to 49	21%	33%	19%	26%
	50 to 249	12%		7%	
Large companies	250 to 500	4%	22%	5%	25%
	501 to 1000	4%		9%	
	1001 to 5000	8%		2%	
	5001 to 10'000	2%		2%	
	10'001+	4%		7%

**Table 3:** Department of interview and survey respondents

Respondent department	Survey respondents (*n = 155)*	Interview respondents (*n = 43)*
General management	39%	53%
Sustainability and CSR	20%	30%
Marketing and sales	15%	5%
Research and development	12%	12%
Production	8%	-
Other	6%	-

Using the statistical classification of economic activities in the European Community (NACE) [47], companies were asked to indicate in what sector they perform their primary business activities. Though the second largest group in Table 4 was the category “Other service activities”, which is mainly designated for repair services [47], it became evident, after analysing the answers of individual responses, that some companies in this category were in fact consultancy firms. According to the NACE subcategories of industry sectors, consultancy activities should be classified under the sector “Professional, scientific and technical activities”. This measurement error [44] was taken into account in the further analysis of the results by interpreting the answers as coming from consultancies. As in the survey, the largest group of the interviewees were active in the manufacturing sector, whereas consultancies (“Other service activities” and “Professional service activities”) took the second spot. Overall, the results are thus representative for a large variety of sectors, with a focus on those sectors primarily associated with CE practices [29, 48].

**Table 4:** Company sectors of interview and survey respondents

Company sector	Survey respondents (*n = 155)*	Interview respondents (*n = 43)*
Manufacturing	27%	21%
Other service activities	24%	16%
Professional, scientific and technical activities	10%	14%
Water and waste management	10%	7%
Construction	7%	10%
Other	22%	16%
Accommodation and food service activities	Incl. in Other (<7%)	9%
Information and communication	Incl. in Other (<7%)	7%

A list of interviewees and the attributes of their companies (department, company size, country and sector) is provided in Table 5 of Appendix 1.

### Survey Development

The survey was drafted according to the seven-step framework for social scientists by Gideon [49]. It contained 22 close-ended questions and one open-ended question to standardise the questioning process and took an average of 25 min to complete. Special care was attributed to the fact that it was an online self-completion questionnaire, sent out with a personal email invitation [50]. It was developed in a participatory way, involving seven researchers, two private partners of the research project specialised in sustainability and life cycle-based assessments and companies engaged with CE practices. Thereafter, the survey was translated from English to Italian and Dutch, tested in all three languages by four large multi-utility companies, a production firm, and two coordinators of CE networks and then sent out to 809 companies.

The survey questions covered in this paper mainly cover the companies’ understanding of the CE concept and the link between CE and sustainability. To answer the two survey questions (available in Appendix 2), first, the respondents indicated the level of importance they attribute to seven CE characteristics, identified from [8, 11, 12, 16, 20, 43, 51]. It was also possible to add additional characteristics in an open text field as to extend the scope of potential answers. Second, they provided their level of agreement with six statements connecting CE and sustainability, the latter of which was expressed as the SDGs and the TBL concept. The answers to both questions were captured on a 5-point Likert scale [49].

This article sets out the first main topic of how companies are connecting CE and sustainability. Upcoming publications will discuss the remaining survey questions, including CE and sustainability assessment, and the goals of CE practices.

### Interview Process

In order to better understand how frontrunner companies engaged with CE practice understand the differences and similarities between CE and sustainability, the authors conducted 43 semi-structured interviews administered via video calls, each with a duration between 45 and 90 min. The semi-structured format allowed the interviewers to ask each interviewee the same questions, while providing room to clarify and contextualise certain issues [41]. The interview guidelines were developed after analysing the survey results and broadly covered the main topics outlined in the previous section. This article analysed the answers to the set of questions (available in Appendix 3) regarding the link between CE and sustainability. Respondents were asked for their own definition of both CE and sustainability, and whether companies should strive for CE, sustainability or both. Moreover, since respondents had previously raised the concepts of bioeconomy and sufficiency within the open comment section of the survey, they were also asked whether they thought the bioeconomy and the idea of sufficiency were relevant to CE. Depending on the interviewees’ preference, the interviews were held in Dutch, English or Italian, with one of the three interviewers. Each interviewer held the interviews in one language only. Therefore, the authors opted for Loubere’s Systematic and Reflexive Interviewing and Reporting (SRIR) method [52], instead of writing full transcripts. This method requires scholars to hold frequent meetings to discuss the findings and impressions of the individual interviews. Hence the interviewers held weekly calls to talk about the main insights and to attune their interpretation of the interview guidelines, thus reducing interviewer variability [53]. Furthermore, the interviews were recorded and the interviewers took notes during the interview process. Thereafter, the interviewers listened to the recordings and complemented their notes, where necessary, to keep interviewer-related errors to a minimum.

### Data Analysis and Integration

The qualitative data analysis employed in this study is based on thematic analysis [54]. Once the survey was closed, all survey data was exported from SurveyMonkey into the statistical analysis software IBM SPSS Statistics 26 [55]. Here, the qualitative information was coded into numerical variables. A univariate analysis approach was taken and frequency tables were created for each variable explored. Subsequently, the authors performed descriptive statistical analyses and cross-tabulations to the relevant dataset to investigate whether differences in the responses could be ascribed to the country of respondents. Given the almost equal distribution of Italian and Dutch companies in the sample, the selection bias could be expected to be minimal. Besides analysing the descriptive statistics results including the mean and standard deviation in more detail, Fisher’s exact test, suitable for small sample sizes, was applied for the cross-tabulations, given that more than 20% of the answering options had frequencies < 5 [56]. Since the questions were based on a 5-point Likert scale with a midpoint, the values could not be aggregated to either the positive or negative side of the scale, which might have led to more significant results. Therefore, the authors used the interviews to further investigate and substantiate these tendencies.

**Fig. 1:**
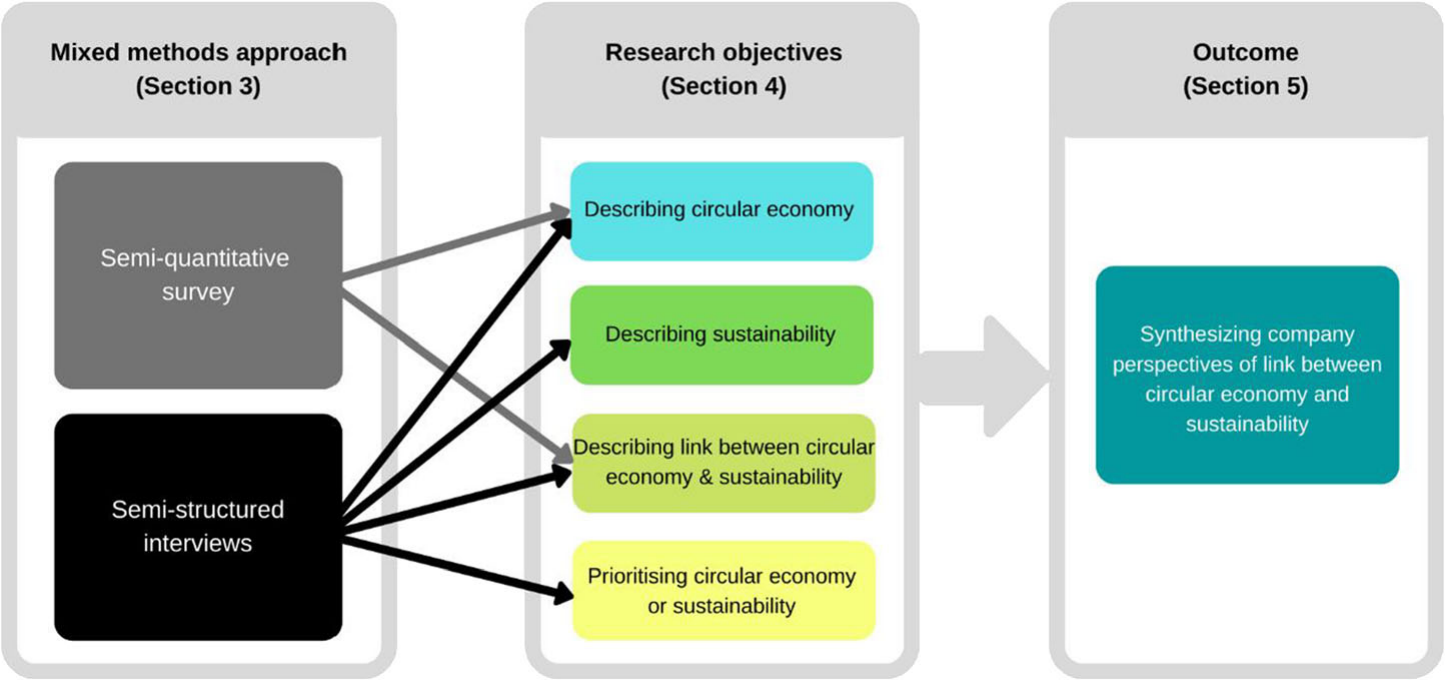
Research design matching the mixed methods approach with the research objectives

Regarding the interviews, all data, including the respondent attributes such as company size, sector, country and position of interviewee, was imported into the qualitative data analysis software NVivo R1 [57] in the English language. Thereafter, the 43 responses were analysed question-by-question using a thematic analysis coding system according to Braun and Clarke [54]. This coding system was created and employed jointly by the three interviewers to reduce both intra-coder variability and inter-coder variability [53]. Using “open coding”, participant responses for each interview question were assigned codes which were later grouped, compared and transformed into themes. It needs to be noted that participants’ responses could be assigned to several different codes on the same topic, which is why the number of respondents per question is only roughly indicated in the result section. Finally, the results from the survey were compared and integrated with the results from the interviews, as seen in Figure 1, which illustrates the overall research design.

## Results

The following subsections integrate answers from both the survey and interviews according to the four research objectives as illustrated in Fig. 1: describing frontrunner company perceptions of CE and sustainability, describing how they understand the link between CE and sustainability and describing how they prioritise these two concepts*.*

### Describing Circular Economy

**Fig. 2:**
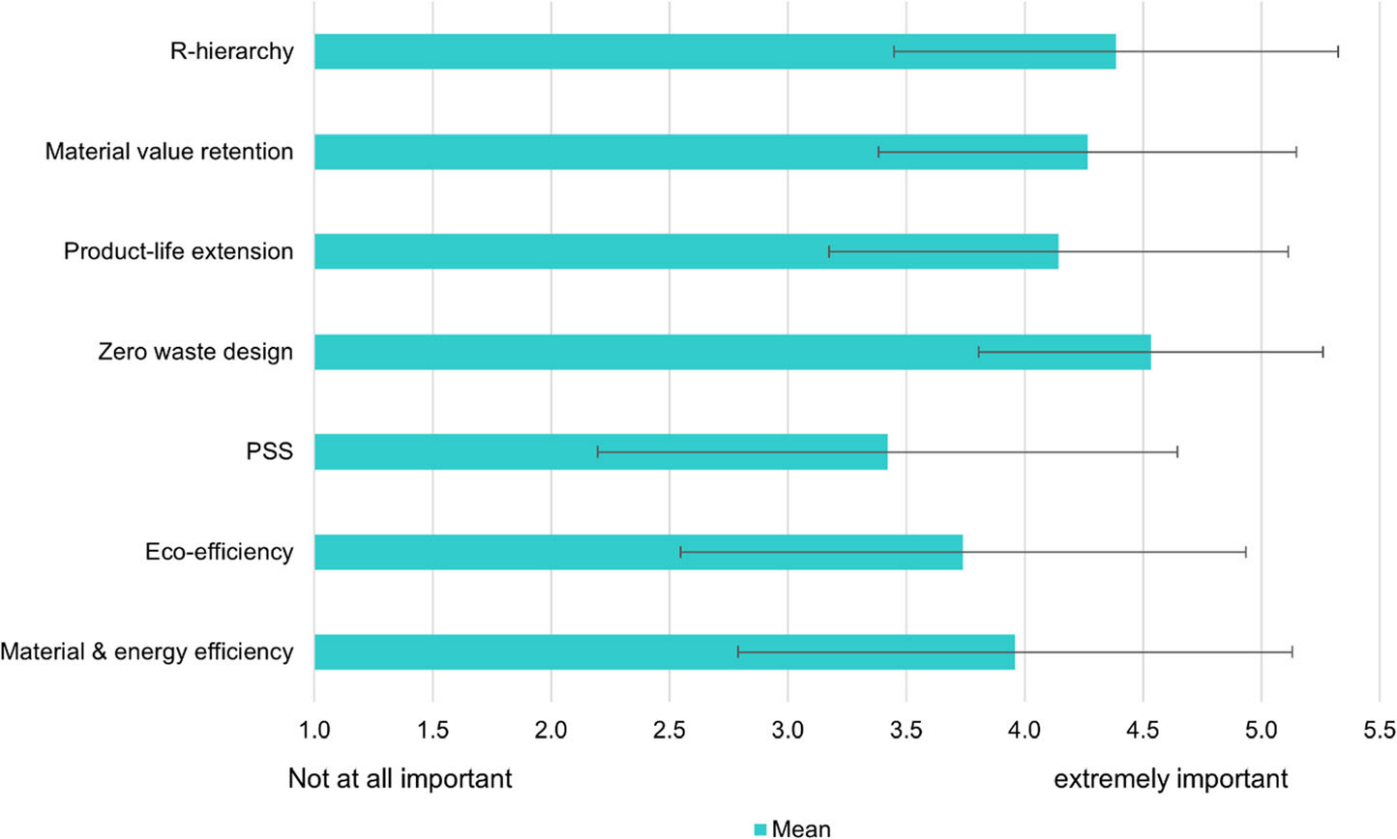
Respondents’ understanding of CE concept, assessing the importance of characteristics with scale from 1 (not at all important) to 5 (extremely important). Standard deviations are represented by error bars. “I don’t know” responses were excluded from mean and standard deviation, *n=*155

Figure 2 presents the first survey question with seven CE characteristics to which respondents were asked to attribute some degree of importance (full statements, standard deviation and statistical significance are available in Appendix 4). Overall, the high consistency between responses, indicated through a low standard deviation, points towards a consensus of the CE concept amongst frontrunner companies. Furthermore, there were only two cases of statistically significant (at *p*<0.001) differences between the answers of Dutch and Italian companies, substantiating the argument of similar perspectives across countries. The fourth statement “Products are designed in a way that eliminates waste” was identified as having the highest level of importance when characterising CE, though all seven characteristics are seen as rather important. It also appeared that the statement ranking second, underlining the importance of the “R-hierarchy”, was perceived to be significantly more important in the Netherlands than in Italy. Interestingly, companies were least likely to characterise the concept of CE with the statement describing businesses shifting towards offering Product Service Systems (PSS). Also, eco-efficiency was perceived to be a slightly less important characteristic than material-efficiency when defining CE, though responses for both characteristics had a high standard deviation. Concerning eco-efficiency, there was another statistically significant difference between the two countries, with Italian companies assigning it more importance. Besides the seven characteristics mentioned, several survey respondents acknowledged the importance of the bioeconomy and the concept of sufficiency to the characterisation of CE within the additional comments field.

After finding the survey respondents generally agreed on the importance of the prescribed CE characteristics, interview respondents were asked how their company would describe CE in an open-ended question. Only few companies directly quoted well-known CE definitions (e.g. from EMF [58] or the EU Action Plan [15]); the majority of companies answered with self-adapted definitions of CE specific to their own context. Frequently, descriptions were supported with practical examples demonstrating their company’s CE implementation.

The interviewed companies most often described CE as a set of activities focusing on resource management at an operational level. This was illustrated through the frequent mention of several related topics including “value retention”, “closing the loop”, “waste prevention” and “material use”. Responses mentioning “value retention” often described CE using some of the 10 R-strategies [43] as a way to both valorise waste and facilitate value creation. Responses that referred to “closing the loop” often indicated that for their company, CE activities guaranteed that resources were available and part of multiple-use cycles. Some companies noted that CE activities should not only avoid the use of non-renewable resources but also prevent waste generation: “CE only makes sense if it adds value, it should not be implemented if more energy is required or more materials are going to landfill” (interviewee #40). A smaller subset of companies that did not refer to resource loops still highlighted waste prevention activities and material use as critical elements of CE, mentioning the terms “zero waste” and “material-efficiency” specifically.

Almost half of the companies described CE as “integral to their business model so it’s not really an option to exclude or sacrifice” (interviewee #32). Rather than only listing CE activities, companies explained how CE was becoming increasingly intertwined with CSR and their corporate strategy; as interviewee #26 said: “It is important to be part of the CE market […] we believe there is a real market opportunity.” CE activities provide opportunities for innovation and these companies acknowledged the competitive advantage and enhanced corporate image of utilising CE as a strategic management tool.

One-quarter of companies illustrated how CE promotes societal change and, for them, a “mindshift” to a new way of conducting their business activities. Interestingly, a few companies, predominantly micro-companies, linked CE activities specifically to respect for nature and experiencing an increased sense of stewardship over natural resources.

Over a third of all companies reflected on the evolving discussions attempting to define CE, with many suggesting that the term CE was quickly becoming another “container concept”. This allowed for the inclusion of many different terms under the umbrella of CE and eventually led to a multi-interpretable and therefore increasingly bland concept. Several companies considered that CE was context-dependent and within their companies, finding one definition was still and would always be a work-in-progress, due to the flexible nature of CE. Interviewee #8 explained: *“*If we are asked what the CE is, we always adapt our answer to the respective company, as the realisation of CE is different in every firm.”

In order to explore the connection of CE with the bioeconomy and the concept of sufficiency raised within the survey results, interview participants were asked whether these are relevant concepts within the CE discussion. Regarding the concept of sufficiency, more than half of the interview participants agreed that sufficiency was an important part of CE. The main reasoning for this was CE’s potential influence on reducing the quantity and improving the quality of societal consumption. In this respect, interviewee #1 argued: “Sufficiency is very important […] in our shop it is possible to buy exactly as much as you need and use only what you need, not determined by standard packaging sizes.” However, the interviewees did not agree on whose responsibility it was to follow a sufficiency-based consumption approach. A few companies that considered sufficiency relevant for CE stated that it was not their responsibility to encourage this behaviour, but the responsibility of consumers, whose demands were prioritised. Others felt that they had little influence on the behaviour of consumers due to their business-to-business sales models, limiting their contact with consumers. On another note, the companies which stated sufficiency was not relevant for CE suggested that separate discussions on consumption should focus on encouraging more responsible consumption habits rather than simply telling consumers to purchase less. A similar trend emerged with interview responses concerning the concept of bioeconomy, with the majority of interviewees agreeing that bioeconomy is considered relevant to CE. Bioeconomy was most often described with reference to the biological cycle of CE, the regeneration of materials and the availability and selection of renewable resources. Similarly to responses regarding sufficiency, some companies declared that the concept of bioeconomy was not relevant or applicable within their scope of operations. Others raised the issue of bioeconomy circularity trade-offs, namely, that some biobased solutions were not inherently circular, as stated by interviewee #5: “Bioeconomy is not necessarily the same as CE since there are biobased materials that are not biodegradable, while there are also synthetic, biodegradable plastics which are not biobased.”

### Describing Sustainability

The topic of sustainability was not included explicitly in the survey which focused on CE. After reviewing the survey results, the authors addressed the concept of sustainability in the interviews in order to get a better picture of the connection between CE and sustainability.

When companies were asked how they describe sustainability, distinct connections with existing sustainability theory were made by around one-third of all companies. Within this group, the three-pillar conceptualisation of (social, economic and environmental) sustainability was mentioned most frequently, sometimes alongside the notion of the TBL framework [38] or, as it is more commonly known, as “People, Planet, Profit” (PPP), coined by the same author. The well-known sustainability definition from *Our Common Future*, or the Brundtland Report [59] also emerged a few times in this context. Similar intergenerational aspects were heard in interview responses that considered sustainability to be closely associated with future generations, or with the future of the planet. These answers centered around topics such as continuity, durability, the capacity to continue certain production activities throughout time, and environmental stewardship. Some participants saw this as an essential aspect of being able to continue their activities, as elucidated by interviewee #37: “If we would now ignore negative environmental impacts, we could not do our work anymore in the future.”

A prominent trend throughout the answers was found in the plethora of examples considering the social pillar of sustainability. These examples can be divided into several categories, with the following occurring most frequently: “community” (and territorial perspective), “well-being”, “job creation”, “employees”, and “human behaviour”. Notably, less examples were provided that could be attributed more directly to the environmental pillar of sustainability, such as a reduction of carbon emissions. This might have been caused by the order of the questions, causing companies to contrast or build upon responses to the previous question about their interpretation of CE. In a few cases, examples of material-efficiency related matters were provided as an illustration of what sustainability meant to the participants’ organisations: value retention, end-of-life insights and regeneration of resources emerged in this category.

When describing sustainability, around one-third of the participants included wide-ranging sustainability practices from avoiding toxic materials, to installing solar panels. Interestingly, a smaller share highlighted that sustainability forms the (strategic) core of their activities. This is similar to the previous finding of companies indicating that CE was integral to their business. In some cases, sustainability was considered a prerequisite to handle the previously named future challenges, given “[sustainability] has been integrated for a long period of time and companies are now feeling comfortable with embedding it within their organisations to ensure longevity” (interviewee #10). In other cases, institutional conditions combined with idealistic motives were the reason for strongly embedding sustainability principles into the organisation, as described by interviewee #34: “When a company has a good approach in terms of sustainability (3 pillars), it is a company that can reduce their risk and can improve the value of the company to investors and stakeholders and take care of the community and employees.”

General criticism of the term sustainability also emerged. Again similar to CE, a few participants considered sustainability to be a “container concept”. These companies also indicated that a single definition did not exist, and a small number of participants considered the term sustainability to be “overused”: “Sustainability is often used as a concept or term, while it is not entirely clear whether the claims can actually be substantiated” (interviewee #11).

### How Companies Connect Circular Economy and Sustainability

**Fig. 3:**
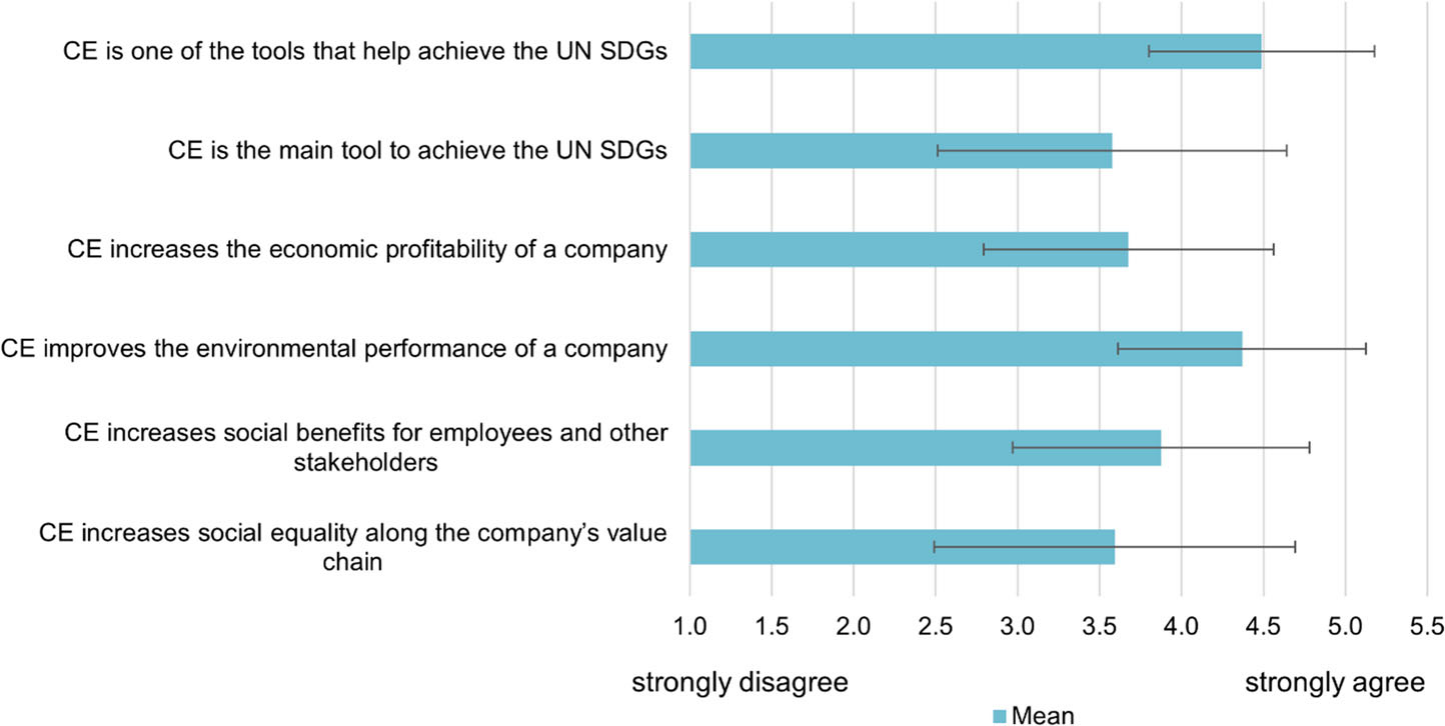
Respondents’ understanding of the link between CE and sustainability, indicating level of agreement with a scale from 1 (strongly disagree) to 5 (strongly agree). Standard deviations are represented by error bars. “I don’t know” responses were excluded from mean and standard deviation, *n*=155

Within the survey, respondents were asked to indicate their level of agreement with six statements describing the effect of CE on sustainability represented as the SDGs and the TBL. The results in Figure 3 indicate that respondents agreed that the concept of CE had a positive relationship with all three sustainability pillars. More specifically, most survey respondents concurred that CE was *one of the tools* to help achieve the SDGs, while they did not necessarily agree that it was the *main tool* to achieve them. Answers further showed that the association of CE with the environmental pillar of sustainability was the strongest, followed by social benefits and economic profitability. Interestingly, it became apparent that respondents agreed less strongly that social equality was increased along a company’s value chain when implementing CE practices. Concerning the statistically significant differences, it is further relevant to point out that Dutch companies were significantly (at *p*<0.001 and *p*<0.05) more likely to disagree with the fourth and fifth statements concerning the social dimension. Furthermore, visible from looking at the standard deviation, the second and fifth statements were the most contested (more detail in Appendix 4).

One open-ended interview question was designed to explore the motivations of respondents when considering implementing CE to improve their environmental performance and how this and the other two pillars influence the organisations’ decision-making processes.

The most frequently mentioned motivation, indicated by more than half of the interviewees, showed that their choice for CE practices was indeed primarily motivated by CE’s perceived positive impacts on the environmental pillar. Notably, only 5 of these interviewees were a part of large companies. Zooming in on this group of answers, more nuanced reasons for this environmental focus emerged. According to some respondents, the legitimacy of measuring environmental impacts as compared to economic and/or social impacts played an important role, e.g. internationally recognised environmental targets as stipulated by the Paris Agreement. A few companies justified the implementation of their CE practices for environmental reasons by referring to the well-established nature of environmental sustainability. They stated that the presence of e.g. international agreements has positively influenced the general level of understanding of environmental sustainability and made it a priority for their clients. Another motivation was provided by a smaller group of interviewees who indicated that environmental benefits derived from CE implementation would, in the long run, also bring about social and economic benefits. The third most frequently mentioned motivation, by a quarter of the respondents, all of which were SMEs, considered this finding to reflect the idealistic motivation of frontrunner CE companies. In the words of interviewee #29: “They are visionaries and have a goal that is bigger than just finances.” Interestingly, some interviewees showed doubts about the underlying reasons for companies implementing CE practices found in the survey. They highlighted organisations could potentially use CE for “greenwashing” because of its associated—but not necessarily proven—positive environmental impacts.

In contrast to the previous motivations, half of the interviewees underlined that the main drivers to engage with CE were still rooted in ensuring economic performance and efficiency. Again, views were diverse within this group. One-third indicated that a stronger economic performance would introduce environmental and social benefits, with companies stating that a profitable business model was considered a requirement for operation and a necessity to be able to achieve sustainability objectives. Additional reasons for pursuing CE with primarily financial motivations were provided by interviewees: to satisfy their clients’ focus on costs and to make use of external incentives, such as governmental grants for funding new innovative CE solutions.

The economic and environmental pillars of sustainability were often considered to go “hand-in-hand”, so the proposals for CE implementation were easier to communicate, illustrated by interviewee #27: “The high-value reuse of materials also leads to a higher price and therefore to better financial performance, these elements go hand in hand.” The social dimension was, on the other hand, sometimes indicated to be somewhat out of focus to the organisation or considered less appealing in terms of storyline and communication towards stakeholders.

One-third of the responses raised the issue that they considered the three dimensions of sustainability inseparable and equally important in terms of decision-making. They were complemented by participants who indicated that the answer depended on who was asked within the organisation: employees associated with sustainability activities would generally pay more attention to the environmental dimensions, while upper-management and founders would rather base their decision-making process on financial parameters. Lastly, the dynamic nature of the decision-making process was highlighted by some who indicated that the mix of context (e.g. the current COVID-19 pandemic), timing, financial maturity and feasibility all influenced which sustainability dimensions would be considered most relevant. The use of “logical thinking” was underlined as well by interviewee #15: “Most decisions are taken based on common sense. A certain direction might seem surprising at first, but it will always be supported by valid arguments.”

It is relevant to note that a quarter of all participants indicated that for them, the social dimension was either fundamental to their decision-making process, or they expected this dimension’s importance to increase greatly in the coming years: “The social dimension will be growing a lot in next 2-3 years; it will be even more important or demanded in the market” (interviewee #34). The companies that attributed high importance to the social dimension were mainly micro “social cooperatives”^1^, of which some highlighted the importance of positively influencing education and citizen participation in the sustainability transition within their decision-making process, and referred to the relevance of “territory” and social innovation to CE practices.

### Should Companies Prioritise Sustainability, Circularity or Both?

When interviewees were asked whether companies should strive for circularity or sustainability, the majority answered they should strive for both. The reasons for this were that while sustainability and CE were perceived as two different concepts in theory, they were closely connected and complementary. Though differences were described as fluid, sustainability was considered a broader concept than CE, as explained by interviewee #23: “Sustainability is larger in scope and I believe that circularity is part of sustainability.” In particular, the social dimension of sustainability was frequently mentioned as a feature that distinguished CE from sustainability, with many interviewees claiming that the people dimension (also including governance and behavioural aspects) was absent from CE. In the words of interviewee #32: “CE would be extremely critical for achieving environmental and potentially economic goals, but CE would not be a direct driver for achieving social goals.” Even so, it was also mentioned that CE covered mainly resource-related aspects, leaving out other environmental aspects such as carbon emissions related to energy and mobility. Yet, some interviewees also said that the difference between CE and sustainability was not yet marked out and that “time will tell. Both concepts are still under development” (interviewee #15). Especially the social dimension of CE still needed to be defined further.

At the same time, CE was perceived as an operational business approach with an economic focus, clear targets and applicable strategies, especially with regard to supply chain management. This was in line with companies’ inevitable focus on cost reduction. Furthermore, CE was said to help maintain companies’ “license to operate”, given that consumption was often viewed negatively from a sustainability perspective. Congruently, the current discourse around this topic was mainly positive and related to entrepreneurship opportunities, while sustainability was sometimes associated with abstention, additional duties, or costs, exemplified by the following quote: “Sustainability has a connotation of ‘it should be less’. This can be a bit negative. CE is much more about: what is possible? It is more positive, more entrepreneurial” (interviewee #16). Interviewee #11 further explained: “In a circular business model you most often solve several problems at the same time, therefore creating more value, making you more competitive and interesting.” Related to the applicability to business, another reason was the more tangible nature of CE. While sustainability seemed too broad of a topic for companies at times, CE was perceived more concrete and “logical”, implemented through Key Performance Indicators (KPIs) already known to companies, such as material and energy use, making its measurement more straightforward.

Moreover, almost one-third of all companies explicitly mentioned that sustainability needed to be the overall goal. Interviewee #38 explained: “CE is a driver for sustainability, a way towards it, so companies should strive for sustainability and use CE as a way to progress towards and also operationalise their sustainability goals.” Whereas there were a few respondents of the opinion that CE would always lead to sustainability, there were also several which pointed out CE was not the only path to sustainability. Thus, for them, sustainability should remain the overall goal of company actions. If circularity were pursued, interviewee #30 mentioned that “a product may be more circular, but from this perspective alone some parts may be produced using child labour.” It thus became clear that the “CE trend”, as it was phrased by interviewee #6, would not replace sustainability, given its longer establishment and wider scope.

A quarter of all companies mentioned that it did not matter whether to strive for circularity or sustainability, because these concepts were in constant flux. These companies said the manifestation of either CE or sustainability practices in a company was highly context-dependent and thus attempting to pin down if a business practice was either sustainable or circular was unrewarding. Interviewee #15 asked: “Why should we talk about the difference at all? It is more important what is done.” An interesting aspect was that firms with this perspective were mainly consultancy companies. As interviewee #16 stated: “It is important that the activities to ‘make the world a better place’ or ‘do more good than harm’ are in the core of the business. This is more important than whether this happens under the CE or sustainability umbrella.” This was also supported by respondents who argued that CE and sustainability were “basically the same” and often “used interchangeably” in companies. Interviewee #39 further said: “CE is actually the new sustainability.” For the few respondents who uttered this position, the concept of CE was merely a subsequent development stage of the sustainability concept, which had been introduced several years before CE. Notably, these interviewees indicated that for them, CE covered all three sustainability dimensions.

## Discussion

**Fig. 4.**
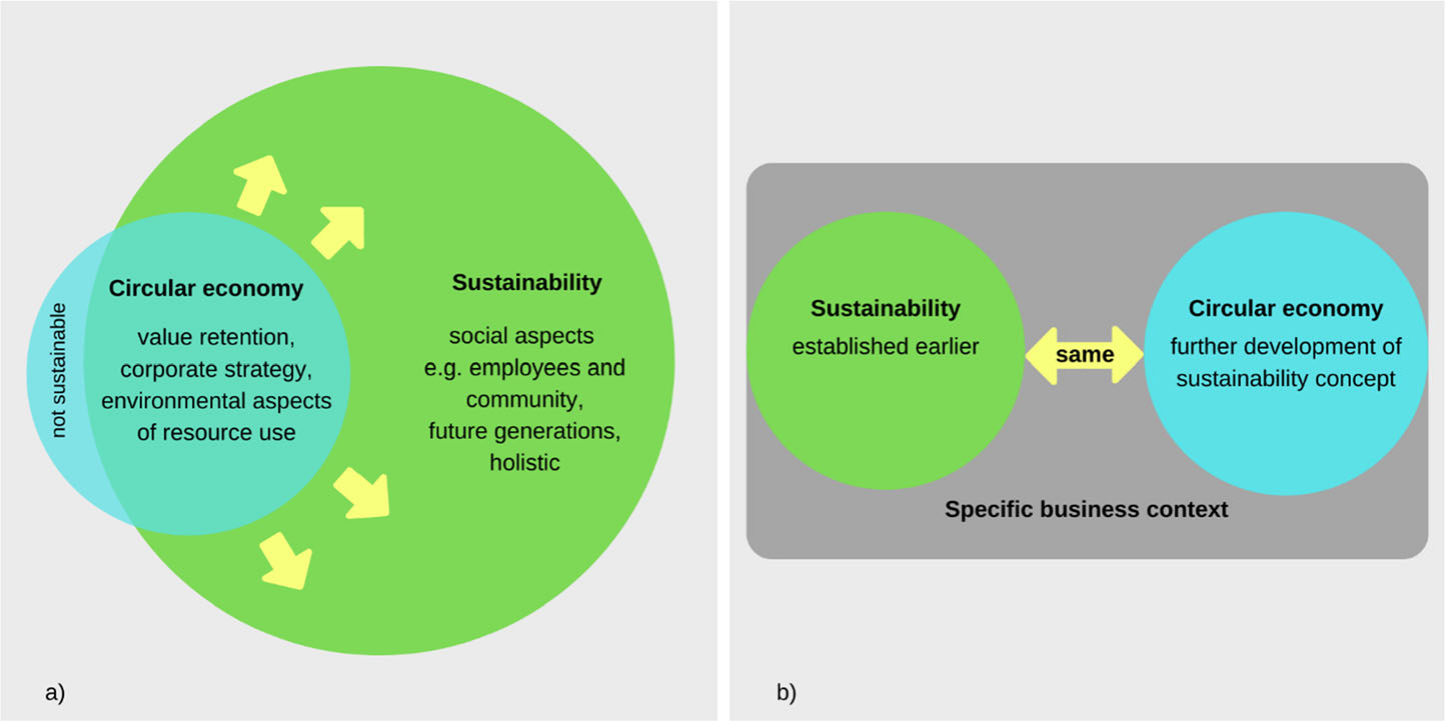
Perspectives of respondents: **a** CE is implemented to achieve sustainability (left). **b** Difference between CE and sustainability is not important (right)

The results have identified two non-mutually exclusive perspectives, as seen in Fig. 4, about the connection between CE and sustainability which were most frequently described by respondents. The first perspective seen in Fig. 4a is that CE is implemented to achieve sustainability, but sustainability is wider than CE. The concepts of CE and sustainability are depicted utilising the themes differentiating them, captured within the interviews. The second perspective, illustrated in Fig. 4b is that the difference between CE and sustainability is not important, as CE and sustainability are the same in practice. Furthermore, sufficiency and bioeconomy emerged as additional concepts associated with CE, through the input of some survey respondents. It was also found that the perspectives on CE and the connection to sustainability did not vary greatly between the two countries, except that for Dutch companies, the R-hierarchy seemed to be a more important concept with regard to CE, which could be linked to popular publications such as Potting et al. [60]. Meanwhile, Italian companies attributed higher importance to eco-efficiency. In the following subsections, the results are first discussed in connection with the two perspectives, after which reflections on the connection between CE and sustainability from a SD perspective are offered.

### CE Is Implemented to Achieve Sustainability

The dominant perspective is that CE and sustainability are different concepts which are closely connected: CE is implemented as a pathway to achieve sustainability. Companies of all sizes and sectors indicated that CE can contribute positively to sustainability impacts. In particular, CE was seen by a majority of the survey and interview respondents as an important way to achieve the SDGs, but not the only one. While different views were encountered, generally, companies use CE to achieve sustainability, without having circularity as their end goal. This is in line with [17] who see CE as a “means to an end”, or “toolbox”, instead of an end in itself, indicated by the yellow arrows leading CE towards sustainability in Fig. 4a. The reason cited by interviewees was that sustainability was more comprehensive than CE, especially with respect to the social dimension. From the survey it also emerged that Dutch companies, in particular, did not expect CE practices to necessarily improve social aspects, in line with the findings of Boons et al. [61], documenting environmental and economic motivations for CE practices. Therefore, CE could not be the only tool to achieve sustainability, as it does not cover the three dimensions to the same degree. Instead, CE is described as more operational, practical and compatible with existing for-profit business strategies and a focus on resources. This is in line with previous findings from CE literature, such as in Schöggl et al. [28] who describe that CE and SD tend to form a subset relationship as depicted in Fig. 4a: while economic and environmental issues are addressed, social topics remain underrepresented, and few solutions on how to incorporate social and consumption-based issues are offered. Yet, several respondents indicated that the difference between CE and sustainability was still under development and that especially the position of the social dimension still needed to be better delineated. First attempts at this were made in e.g. Padilla-Rivera et al. [62] and Walker et al. [63]. It must also be noted that despite numerous authors presenting new definitions for CE [8, 64], companies did not necessarily refer to them. Instead, most interviewed companies described CE using self-adapted definitions specific to their own context, which overlap partially with the CE characteristics presented to the survey respondents. Interestingly, the PSS concept, which was seen as the least important CE characteristic in the survey, was also hardly mentioned in the interviews. This shows a contrast with academic literature, where PSS is often described as a CE opportunity [65, 66].

Moreover, the results by Schroeder et al. [17], describing that the connections between CE and several SDGs range from weak to strong, were partially reflected in the findings of this research. While the survey results showed CE was not found to be a promising tool to decrease inequality in the supply chain, it was seen as a valid pathway for addressing environmental issues. Reasons mentioned in interviews were the readily available assessment tools and metrics for environmental sustainability, as well as its popularity in the market and amongst consumers. Reviews of assessment approaches to CE at the company or micro-level also found that the environmental and economic domains are included more frequently in CE metrics [25, 67]. In fact, the lack of a holistic approach to SD and, more precisely, the neglect of equality in CE, has been described as a long-standing shortcoming of CE research [8, 28]. Some researchers advocate for a more strongly politicised conceptualisation of CE, more distinctly focused on tackling the systemic socio-ecological challenges of the Anthropocene [13]. Related thereto, results further showed most companies did not pursue sustainability dimensions in isolation, but often saw them as interdependent factors. Therefore, decision-making was mainly characterised with a focus on the economic implications leading to impacts on the environmental and at times the social dimension, depending also on the degree of idealism present within the corporate culture.

Additionally, about a quarter of the interview participants noted that there could be trade-offs between CE and sustainability impacts. In Fig. 4a, the trade-offs are indicated by the area of CE labelled “not sustainable”, where CE practices with potentially non-sustainable outcomes are captured. Contextualising this result, the authors refer to Geissdoerfer et al. [10], who identified eight types of relationships between the two concepts discussed in academic literature. While several types of such relationships were discussed throughout the interviews, the trade-off relationship was the second-most mentioned after the beneficial relationship. Following this, it is important to note that the recognition of “CE trade-offs” would alter the perceived relation between CE and SD and therefore, substantiate the need to accurately assess the effects of CE solutions before their implementation. Since most CE practices do not specifically address the social dimension, it is especially important to not turn a blind eye on the potential CE effects on social issues, which was acknowledged by several interviewees.

### Difference between CE and Sustainability Is Not Important

The second perspective that surfaced, shown in Fig. 4b, was that not all participating companies considered pinpointing the conceptual difference between CE and sustainability to be a priority, neither for themselves nor for academia. This mainly emerged from the interviews, in which many companies indicated both CE and sustainability could be considered either: “container concepts”, constantly undergoing change and serving as an umbrella for different practices, or “dynamic concepts” with different meanings in various business contexts. According to several respondents, consisting mainly of consultancy companies, addressing existing barriers to CE, or CE assessment, would benefit companies to a greater extent than discussing semantic differences. Interestingly, in academic literature, the discussion on the conceptual complexities of both concepts and their relation has received much attention (see e.g. [10, 18]), indicating that a considerable divergence might exist between practice (i.e. company needs) and science. Kirchherr and Van Santen [22] have also identified this gap in their critique on the CE research field, highlighting that “practitioners don’t care about the definitional nuances of CE; they want empirical work that provides evidence on how to make CE work” (p. 1). About half of the companies with this perspective also stated that no difference between the concepts existed in practice, because they saw CE as an evolution of sustainability. These respondents also viewed CE as inherently sustainable and covering all three sustainability dimensions. Therefore, they were indifferent to the question whether to strive for CE or sustainability, as it depended on their respective business context.

### Companies’ View on Sufficiency and Bioeconomy

While sufficiency has been described as counterintuitive to the traditional way of doing business, most interviewed companies were aware that it is part of the CE discussion. After all, the first 10-R strategy defined by Reike et al. [51] is “R0 = refuse”. This contradicts the finding that several companies saw CE as a commercial opportunity. Yet, many directed the attention to the agency of consumers, stating that they were the ones that should abstain from consumption. Only a few companies integrated a moderate sufficiency-based CE approach into their business, by ensuring that production or supply would not supersede demand or need, as anticipated by Bocken and Short [36]. With regard to bioeconomy, the majority of companies were quick to recognise the connection of the biological cycle within CE, detailed within the popular butterfly diagram [58]. However, though the bioeconomy is conceptually integrated into CE [33, 58] and supported by international policies [68], some participants were less likely to see the relevance to their business operations. Conversely, attention was awarded to the importance of renewable resources and the principle of cascading as part of CE; this integration and its potential contribution to the SDGs has been examined by Campbell-Johnston et al. [69] in detail.

### Implications for Research and Practice

While previous studies appear to often focus on single sectors, such as manufacturing, the results here show that companies engaged with CE are found to be active in a number of sectors, requiring further cross-sectoral studies [29, 30]. To have a positive impact towards SD, CE practices are ideally implemented with the goal of being sustainable, rather than being circular in itself. However, this article shows that the motivations of companies to implement CE do not necessarily consider the impacts on all three sustainability dimensions. Depending on whether companies took the perspective of CE designed as an enviro-economic model—the *Circular Economy discourse*—or the perspective of CE being no different from sustainability—related to the *Circular Society discourse*—different implications for scientific research follow [13]. In the first case, it is recommended to include the social dimension in the corporate decision-making process; the social dimension can be addressed with existing CSR initiatives [62, 63], while in the second case, specific CE-related social issues are expected to find a place in decision-making frameworks [70]. Rather than providing lip service to the new “trend” of CE, one role of scientists would be to support companies in their pathway towards SD by not merely identifying barriers but also providing solutions to overcoming them. In this context, focusing on established sustainability assessment of CE practices can make CE-sustainability trade-offs visible [71] and allows for promoting CE only when it bears positive sustainability effects.

Acknowledging the respondents’ concern that academics should not continue to focus on defining the differences between CE and sustainability, the authors find it crucial that companies engaged with CE evaluate their underlying motives of implementing CE practices. It is recommended that CE strategies are formulated to positively impact all three sustainability dimensions in a systemic manner. Without this internal evaluation, companies run the risk of reducing opportunities to seize the transformative potential of CE, or of creating adverse sustainability impacts. After goal-setting and considering different CE strategies, assessment approaches can be applied to assess which CE solution is preferable, be it on a qualitative or quantitative level [72]. Given that the results indicated that micro-companies (regardless of their legal form) were more likely to implement CE to achieve broader sustainability goals, the development of holistic assessment should consider the capabilities of micro-companies. Companies engaged with CE can further explore sufficiency-driven business strategies, for which Khmara and Kronenberg [73] have proposed seven indicators for firms to self-assess their operations. The advancement of sustainability assessment approaches for CE practices enables companies to externally report the impacts of their CE practices in response to increasing demands for transparency from stakeholders and to demonstrate alignment with existing and developing governance frameworks e.g. the SDGs and the new Action Plan on CE under the European Green Deal [68]. While this degree of accountability for CE practices could be seen as cumbersome, diligent reporting will provide the foundation to receive CE-related funding and will offer new business opportunities with like-minded professionals.

## Concluding Remarks

Using a mixed-methods approach, this article explored how companies engaged with CE practices view the connection between sustainability and CE. Two main perspectives were found: the first suggests that frontrunner companies generally view sustainability to be a wider, more holistic concept, including the social dimension, when compared with CE. At the same time, firms emphasise that CE offers (only) one possible operational pathway to a more sustainable society. The second perspective shows that companies do not consider the identification of conceptual differences between CE and sustainability to be a priority, as for them the two concepts are the same in practice. This shows that for companies engaged with CE, the concepts are closely connected, even though the motivations for implementing CE practices can be diverse. Ultimately, firms were aware that the goal of implementing CE ideally consists of striving for a more sustainable world. The findings presented in this article provide an overview of the relation between CE and sustainability, informed by experiences of companies engaged with these concepts. Eliciting these private sector perspectives makes an empirical contribution to a discussion which has, to date, largely remained in the theoretical realm.

The process of carrying out and analysing the survey and interviews comes with various limitations. These mostly relate to extrapolating the results to organisations outside of this sample, which is composed of highly diverse frontrunner companies engaged with CE across different sectors, sizes and CE activities. Moreover, the sample contains a large share of micro-companies, which have distinct ways of operating when compared to larger firms. However, as was observed in both the survey and interviews, company size did not have a significant impact on the perspective of companies on the CE and sustainability connection. Furthermore, the study exclusively covers firms operating in Italy and the Netherlands, which potentially allows for generalisations to Western Europe only. Finally, the interviews were conducted in three languages which could have provided room for translation inaccuracies, although various methods were employed to minimise this.

Given this article focused only on companies already engaged with CE practices, further research to understand private sector interpretations of CE and sustainability could include firms which are new to CE. Future studies could also use the current findings to inform the development of corporate strategies for companies starting with their CE journey, while not letting sustainability out of sight. On the same note, research and practice are recommended to jointly develop impact assessment approaches to support these corporate strategies. Such co-created assessment approaches have the potential to enable firms engaged with CE to steer their transformative potential towards advancing the SDGs.

## Appendix 1

**Table 5:** List of interviewees and their attributes

Code	Department	Company size	Country	Sector
Interviewee #1	General management	Micro	Italy	Accommodation and food service activities
Interviewee #2	General management	Micro	Italy	Construction
Interviewee #3	Sustainability and corporate social responsibility	Micro	Italy	Other
Interviewee #4	Marketing and sales	Micro	Italy	Accommodation and food service activities
Interviewee #5	Research and development	Micro	Italy	Professional service activities
Interviewee #6	General management	Micro	Italy	Other service activities
Interviewee #7	General management	Micro	Italy	Manufacturing
Interviewee #8	General management	Micro	Italy	Professional service activities
Interviewee #9	General management	Micro	Italy	Manufacturing
Interviewee #10	General management	Micro	Netherlands	Other service activities
Interviewee #11	General management	Micro	Netherlands	Other
Interviewee #12	Research and development	Micro	Netherlands	Construction
Interviewee #13	General management	Micro	Netherlands	Other
Interviewee #14	Sustainability and corporate social responsibility	Micro	Netherlands	Construction
Interviewee #15	General management	Micro	Netherlands	Professional service activities
Interviewee #16	General management	Micro	Netherlands	Other
Interviewee #17	Sustainability and corporate social responsibility	Micro	Netherlands	Other
Interviewee #18	General management	Micro	Netherlands	Other
Interviewee #19	Sustainability and corporate social responsibility	Micro	Netherlands	Other service activities
Interviewee #20	General management	Micro	Netherlands	Professional service activities
Interviewee #21	General management	Micro	Netherlands	Other
Interviewee #22	Sustainability and corporate social responsibility	Small-Medium	Italy	Other service activities
Interviewee #23	General management	Small-Medium	Italy	Other
Interviewee #24	General management	Small-Medium	Italy	Accommodation and food service activities
Interviewee #25	Research and development	Small-Medium	Italy	Manufacturing
Interviewee #26	General management	Small-Medium	Italy	Manufacturing
Interviewee #27	General management	Small-Medium	Netherlands	Water and waste management
Interviewee #28	Sustainability and corporate social responsibility	Small-Medium	Netherlands	Other
Interviewee #29	General management	Small-Medium	Netherlands	Construction
Interviewee #30	General management	Small-Medium	Netherlands	Other service activities
Interviewee #31	General management	Small-Medium	Netherlands	Other service activities
Interviewee #32	General management	Small-Medium	Netherlands	Manufacturing
Interviewee #33	Research and development	Small-Medium	Netherlands	Other
Interviewee #34	Sustainability and corporate social responsibility	Large	Italy	Manufacturing
Interviewee #35	Sustainability and corporate social responsibility	Large	Italy	Accommodation and food service activities
Interviewee #36	Research and development	Large	Italy	Water and waste management
Interviewee #37	Sustainability and corporate social responsibility	Large	Italy	Water and waste management
Interviewee #38	Sustainability and corporate social responsibility	Large	Italy	Manufacturing
Interviewee #39	Sustainability and corporate social responsibility	Large	Netherlands	Construction
Interviewee #40	Marketing and sales	Large	Netherlands	Other
Interviewee #41	Sustainability and corporate social responsibility	Large	Netherlands	Manufacturing
Interviewee #42	Sustainability and corporate social responsibility	Large	Netherlands	Other
Interviewee #43	General management	Large	Netherlands	Other service activities

## Appendix 2

### Survey questions in English

1. The circular economy concept is a developing concept and thus not yet clearly defined. According to your understanding, which statements below characterise a circular economy?
  *Please assign a level of importance to each potential characteristic of the circular economy.*
  Characteristic not important at all, slightly important, moderately important, very important, extremely important

- During the life cycle of a product, materials are reduced, reused, recycled, or recovered
- Goods are produced in a way that enables the maintaining and recovery of value of materials such as gold and other scarce materials
- Goods are produced, or services are provided in a way that increases their durability, before they are disposed
- Products are designed in a way that eliminates waste, because after their end of life, they re-enter the value chain as material input
- Businesses offer a service to users, instead of selling their products to customers (e.g. renting a car, instead of selling it)
- More goods and services are produced while causing less negative impacts on the environment
- More goods and services are produced while using fewer material resources or energy
- Other, please specify:

2. Circular economy is often mentioned in connection with sustainability. In your opinion, what kind of effect does the circular economy have on the three sustainability pillars (environment, society and economy)?
  *Please indicate your level of agreement with the following statements.*
  Strongly disagree, disagree, neither agree nor disagree, agree, strongly agree

- The circular economy is one of the tools that will help achieve the UN sustainable development goals
- The circular economy is the main tool to achieve the UN sustainable development goals
- The circular economy increases the economic profitability of a company
- The circular economy improves the environmental performance of company
- The circular economy increases social benefits for employees and other stakeholders
- The circular economy increases social equality along the company’s value chain

## Appendix 3

### Interview guidelines

1. How would your company describe circular economy?
  - Several companies have brought up the themes of sufficiency and bioeconomy as an integral part of Circular Economy. Are these concepts relevant to your company as well?
2. How would your company describe sustainability?
  - Do you think companies should strive for sustainability, circularity, or both? Why?
  - Results from our survey suggest that companies were most strongly motivated to implement Circular Economy practices in order to improve their environmental performance over improving their economic or social performance. Why do you think this is the case?
  - How do you integrate (or balance) the three dimensions of sustainability within the decisionmaking process in your company?

## Appendix 4

### Detailed survey results

**Table 6:** Respondents’ understanding of CE concept with survey statements linked to CE characteristics

Statements in survey	CE characteristic	N.^a)^	Mean	Standard deviation	Significance Fisher’s test
During the life cycle of a product (production, use, end-of-life) materials are reduced, reused, recycled, or recovered	R-hierarchy	153	4.4	0.94	0.001**
Goods are produced in a way that enables the maintaining and recovery of value of materials such as gold and other scarce materials	Material value retention	151	4.3	0.88	0.130
Goods are produced or services are provided in a way that increases the durability of products, before their disposal	Product-life extension	154	4.1	0.97	0.190
Products are designed in a way that eliminates waste, because after their end of life, they re-enter the value chain as material input	Zero waste design	150	4.5	0.73	0.474
Businesses offer a service to users, instead of selling their products to customers (e.g. renting a car, instead of selling it)	PSS	150	3.4	1.22	0.113
More goods and services are produced while causing less negative impact on the environment	Eco-efficiency	146	3.7	1.19	0.001**
More goods and services are produced while reducing material resource or energy use	Material & energy efficiency	146	4.0	1.17	0.363

^a)^excl. “I don’t know” responses

*significant at 95% confidence interval

**significant at 99% confidence interval

**Table 7:** Respondents’ understanding of the link between CE and sustainability

Statements	N. a)	Mean	Standard deviation	Significance Fisher’s test
The circular economy is one of the tools that help achieve the UN sustainable development goals	153	4.5	0.69	0.410
The circular economy is the main tool to achieve the UN sustainable development goals	151	3.6	1.06	0.119
The circular economy increases the economic profitability of a company	152	3.7	0.89	0.949
The circular economy improves the environmental performance of a company	154	4.4	0.76	0.103
The circular economy increases social benefits for employees and other stakeholders	146	3.9	0.91	0.001**
The circular economy increases social equality along the company’s value chain	142	3.6	1.10	0.040*

^a)^excl. “I don’t know” responses

*significant at 95% confidence interval

**significant at 99% confidence interval

## Footnotes

1 Social cooperatives are a specific legal form of organisation in Italy and are, by law, founded with the intent to create social, health or educational benefits for their members and the wider society or to stimulate labour market inclusion of disadvantaged people.
